# The Post-COVID-19 Economic Policy Uncertainty and the Effectiveness of Monetary Policy: Evidence From China

**DOI:** 10.3389/fpubh.2021.771364

**Published:** 2021-10-29

**Authors:** Yuegang Song, Yanling Yang, Jianzhong Yu, Zhichao Zhao

**Affiliations:** ^1^School of Business, Henan Normal University, Xinxiang, China; ^2^Institute of International Economy, University of International Business and Economics, Beijing, China; ^3^School of Economics, Tianjin University of Commerce, Tianjin, China

**Keywords:** EPU, monetary policy, LT-TVP-VAR model, China, counter-cyclical

## Abstract

The outbreak of the COVID-19 pandemic has caused an upsurge economic policy uncertainty (EPU). Study on the time-varying effect of EPU is of substantial implication for the central bank in implementation of monetary policy. To empirically investigate the time-varying effect of EPU, the paper considers the shock of the monetary policy implemented by China's central bank on different economic variables including interest rate, output gap, and inflationary gap using the latent threshold time-varying parameter vector autoregressive model (LT-TVP-VAR Model). Data period is chosen to be January 2015 through April 2021. Our findings show that (i) EPU has a significant threshold effect on the shock of quantitative monetary policy instrument and the shock of price-based monetary policy, and that the two types of policy are positively correlated; (ii) the price-based monetary policy instrument has a significant counter-cyclical effect on both output gap and inflationary gap; (iii) relative to the quantitative monetary policy instrument, the price-based monetary policy instrument has a more significant counter-cyclical effect on output gap; and (iv) a higher level of EPU is associated with a more significant monetary policy effect on output gap and inflationary gap.

## Introduction

The outbreak of the COVID-19 pandemic in 2020 has caused tremendous shocks on society. As the pandemic has swept the global economy, many economies including China are faced with growth challenge and structural transition. A large number of research findings have acknowledged that economic policy uncertainty (EPU) can be one of the important factors that exert negative effect on economic growth. Driven by various environmental factors, the level of EPU might vary. As the level of EPU increases, it tends to discourages investment and consumption and make financial institutions reduce business credit, which might result in a higher unemployment rate and slow economic growth. Hence, it is vitally important for the central bank to improve monetary policy effectiveness and reduce EPU as the goals of monetary policy typically include price stability, job creation, balance of international payments, and particularly output growth.

In this context, research on the time-varying effect of economic uncertainty is of considerable policy implications for the central bank in monetary policy. The parameters of a basic VAR model and the variance of the stochastic disturbance terms are assumed to be fixed. By contrast, economic & financial environments and macroeconomic policy in the real world are constantly in change, which implies that the parameters might also be in the process of dynamic change. In addition, economic uncertainty differs from ordinary economic variables in that it is not a constant target of monetary policy, and the TVP-VAR model might not be applicable to the research question of this paper. Hence, the LT-TVP-VAR model might be an appropriate choice as it is not only based on time-varying coefficient estimates but also takes care of heteroscedasticity by assuming time-varying volatility (TVV). This paper introduces latent threshold time varying parameter vector autoregressive (LT-TVP-VAR) modeling to investigate the correlation between EPU and monetary policy in search for answers to questions such as how sensitive the quantitative monetary policy and the asset price-based monetary policy (or price-based monetary policy) can be in response to EPU's shock, whether it is possible to ensure full employment by inhibiting inflation or deflation, and whether it is possible to maintain sustainable growth.

The rest of this paper is organized as follows. Section Literature Review provides a discussion of existing literature. Section The LT-TVP-VAR Model discusses the model and section Data describes the data. Section Empirical Results elaborates on empirical results. Section Conclusion concludes.

## Literature Review

The existing research related to this paper involves primarily the topics on the definition of EPU, the shock caused by EPU, monetary policy adjustment mechanism, and the correlation of EPU with monetary policy. Bloom et al. (1) defined EPU as an economic risk in nature, or more precisely, an uncertainty brought to economies as a result of the failure to precisely anticipate whether, when and how the government would introduce economic policy adjustment. The research line on EPU shock is concentrated on research focuses on such aspects as line of credit, investment, GTFP, BTC/USD. Talavera et al. (2) found that the bank's priority choice in face of increasing economic uncertainty is to reduce loans. Li and Yang (3) suggested that increasing EPU would negatively affect investment activities. Wu et al. (4) find that there is a significant causality from the Twitter-based EPU to the BTC/USD. Song et al. (5) found an increase in EPU was shown to decrease green total factor productivity (GTFP).

There are two rules for monetary policy adjustment to follow: quantitative monetary policy and price-based monetary policy. Mccallum (6) proposed the notion of quantitative monetary policy which refers to monetary policy based on money supply, money velocity, and output gap size. By contrast, price-based monetary policy, referred to as the Taylor rule (7), is based on adjustment in the interest rate as a response to output and inflation. Xu (8) suggested a switch from quantitative monetary policy to price-based monetary policy as the principal measure given the current conditions of Chinese economy. According to Ma and Fan (9), the pandemic exerted a major negative shock on the Chinese economy and structural monetary policy serves to make up for traditional monetary policy in the problem of resource allocation distortion. The existing literature on monetary policy effectiveness covers such topics as comparison of different monetary policy instruments and the effect of monetary policy in different regulatory contexts. Bernanke et al. (10) identified the positive effect of the shock of monetary policy. Silvia and Giovanni (11) used Bayesian local projections to find that the credit transmission channel of monetary policy amplifies economic fluctuation. Zhang and Jiang (12) found that monetary policy adjustment differs in effect across different regulatory situations.

In the existing literature on the correlation of EPU and monetary policy, the VAR model and the Dynamic Stochastic General Equilibrium (DSGE) model are used to study EPU's shock on monetary policy effectiveness. In a DSGE-based comparative analysis of various monetary policy rules, Zhuang et al. (13) found that EPU has only a quantitative shock on monetary policy effectiveness. In an investigation based on a non-linear Interacted-VAR model, Pellegrino (14) studied the correlation between EPU and the Eurozone monetary policy effectiveness and the empirical findings show that EPU weakens monetary policy effectiveness. In an investigation, based on Interacted-VAR model, Jin and Zhang (15) found that EPU undermines the effects of macroeconomic policies.

The shock of EPU on monetary policy effectiveness is manifested through channels of bank credit, investment, consumption, output, and price level. Talavera et al. (16) found EPU a significantly pivotal shock on commercial bank lending, which varies remarkably with bank size and profitability. According to Georgiadis and Mehl (17), EPU slows individual investment and consumption. Xu and Wang (18) identified a prominent non-linear effect associated with monetary policy adjustment that takes the form of a remarkable fall in output and price level as a result of a negative shock on demand.

In spite of the large amount of existing studies on monetary policy and EPU, little research attention has been given to the dynamic interaction between EPU and monetary policy adjustment, particularly the time-varying effect of EPU increase on monetary policy and macroeconomic policy. The paper makes three contributions to the existing literature. First, it introduces EPU as an endogenous variable to study the time-varying effect of quantitative monetary policy and price-based monetary policy on output gap and inflationary gap during a growth in EPU. Second, a comparative analysis of pre-pandemic and post-pandemic monetary policy effectiveness highlights the significant role which EPU plays in monetary policy adjustment. Third, the paper uses the LT-TVP-VAR model to study the dynamic effects of EPU on monetary policy adjustment which provides a new approach to subsequent relevant research from an empirical perspective.

The rest of the paper is organized as follows. Section The LT-TVP-VAR Model introduces the LT-TVP-VAR model. Section Data discusses indicator selection and data processing. The empirical analyses in section Empirical Results cover the parameter estimates, threshold effect test, impulse response analysis, shock of quantitative monetary policy and price-based monetary policy on output gap and inflationary gap, and a comparative analysis of how the policies shock on pre-pandemic and post-pandemic inflationary gaps. Section Conclusion provides the conclusion and policy recommendations.

## The LT-TVP-VAR Model

The traditional VAR model is presented as follows:


(1) $$\begin{array}{l} \Phi y_{t}=p_{1}y_{t-1}+p_{2}y_{t-2}+p_{s}y_{t-s}\\ \;+\mu_{t},t=s+1\cdots n \end{array}$$

Where, Φ and *p* form a *k*-order square matrix and μ_t_ is the k^*^1−D structural shock.

It is assumed that the leading diagonal of Φ (i.e., *k*-order square matrix) is 1 and can be expressed in terms of the following triangular matrix:


(2) $$\begin{array}{l} \Phi =\left|\begin{array}{cccc} 1 & 0 & \cdots & 0\\ {}_{\Phi_{21}} & 1 & \cdots & 0\\ \vdots & \ddots & \ddots & \vdots \\ {}_{\Phi_{\text{k}1}} & \cdots & \Phi_{k,k-1} & 1 \end{array}\right| \end{array}$$

In this condition, the original model can be converted into a general SVAR model (Equation 2). Given that $\Gamma i={\Phi^{-1}}^{*}P_{i}$, Equation (1) will be converted to Equation (3) as follows:


(3) $$\begin{array}{l} y_{t}=\Gamma_{1}y_{t-1}+\Gamma_{2}y_{t-2}+\cdots +\Gamma_{s}y_{t-s}\\ \;+\Phi^{-1}\Sigma \varepsilon_{t},\varepsilon_{t}\;\sim \;N(0,I_{k}) \end{array}$$

Furthermore, we can get the following:


$$\begin{array}{l} \sum \text{=}\left[\begin{array}{cccc} \sigma^{2} & 0 & \cdots & 0\\ 0 & \sigma^{2} & \cdots & 0\\ \vdots & \ddots & \ddots & \vdots \\ 0 & \cdots & 0 & \sigma k \end{array}\right] \end{array}$$

Stack the elements in Γ*i* to form vector γ (*k*^2^s^*^1−D), Equation (3) will be converted into Equation (4):


(4) $$\begin{array}{l} yt=X_{t}\gamma_{t}+\Phi^{-1}\Sigma_{t}\varepsilon_{t},t=s+1,\ldots ,n \end{array}$$

Where, *X*_*t*_ = *I*_*k*_ ⊗(y_t−1_,…,y_t−s_) (⊗ stands for the Kronecker product).

In Equation (4), if Φ_*t*_, γ_*t*_, and Σ_t_ change with time, the elements in matrix Φ_*t*_ can be stacked to form ϕ_*t* =_(ϕ_21_, ϕ_31_, ϕ_32_, ϕ_41_, …, ϕ_*k, k*−1_). At the same time, the matrix of logarithmic stochastic volatility ($h_{t}={\left(h_{1t},\ldots ,h_{kt}\right)}^{1}$) can be used, where h_jt_ = lnσ2 jt, *j* = 1,…, *k*, and *t* = *s*+1,…, *n*.

It is assumed that the parameters of the model are all in random walk as follows:


(5) $$\begin{array}{l} \phi_{t+1}=\phi_{t}+\mu_{\phi t} \end{array}$$

(6) $$\begin{array}{l} \gamma_{t+1}=\gamma t+\mu_{\gamma t} \end{array}$$

(7) $$\begin{array}{l} h_{t+1}=h_{t}+\mu_{ht} \end{array}$$

(8) $$\begin{array}{l} \left[\begin{array}{c} \varepsilon_{t}\\ \mu_{\phi t}\\ \mu_{\gamma t}\\ \mu_{ht} \end{array}\right]\sim N\left[0,\left[\begin{array}{cccc} I & 0 & 0 & 0\\ 0 & \Sigma \phi & 0 & 0\\ 0 & 0 & \Sigma \gamma & 0\\ 0 & 0 & 0 & \Sigma h \end{array}\right]\;\right] \end{array}$$

In Equation (8), Φ_*t*_+*1* is equivalent to (μ_Φ0_, Σ_Φ0_), γ_*t*_+1 is equivalent to (μ_γ0_, Σ_γ0_), *h*_*t*_+*1* is equivalent to (μ_*h*0_, Σ_*h*0_), and Σ_Φ_, Σ_γ_, and Σ_*h*_ are all positive definite matrices. It's assumed that the time-varying parameters are not correlated in shock. Equation (8) is a complete TVP-VAR model. In this paper, the LT-TVP-VAR model is built based on the TVP-VAR model by replacing Φ_*t*_ and γ_*t*_ with *a*_*t*_ and *b*_*t*_, respectively. Then we get Equation (9):


(9) $$\begin{array}{l} a_{t}=\phi_{t}•I\left(|\phi_{t}|\geq d_{a}\right);b_{t}=\gamma_{t}•I\left(|\gamma_{t}|\geq d_{b}\right) \end{array}$$

Where, *I*(•) stands for the indicative function with a value of 0 or 1, and *d*_*a*_ and *d*_*b*_ stand for the threshold levels of the time-varying parameters and the simultaneous equation coefficients, respectively. When the absolute values of the time-varying parameters (Φ_*t*_and γ_*t*_) are greater than *d*_*a*_ and *d*_*b*_, *I*(•) is equal to 1, which means that the variables are not interactive in the model. Only when *d*_*a*_ and *d*_*b*_are equal to 0 does the model have no threshold effect, at which time the LT-TVP-VAR model changes back to the TVP-VAR model.

In this paper, model parameter estimation is based on the Markov Chain Monte Carlo (MCMC) algorithm for the Bayesian model.

Assuming that ϕ, γ, and *h* are subject to normal distribution a priori (i.e., $\mu_{\Phi 0}=\mu_{\gamma 0}=\mu_{h0}=0),\;\Sigma_{0}=\Sigma_{\gamma 0}=\Sigma_{h0}=10\times I,{\left(\Sigma_{\Phi }\right)}_{i}^{-2}\sim Gammma\left(40,0.02\right),{\left(\Sigma_{\gamma }\right)}_{i}^{-2}\sim Gammma\left(40,0.02\right)and{\left(\Sigma_{h}\right)}_{i}^{-2}\sim Gammma\left(40,0.02\right)$.

For Bayesian inference for the LT-TVP-VAR model, the first step is to model MCMC. The samples are derived from a posteriori distribution of the correlated parameters. Therefore, an appropriate sampling method must be selected. Combined sampling is conducted for the remaining parameters ($\phi \;\text{=}\;\left\{\;\phi_{t}\;\right\}{\;}_{t=s+1}^{n};\gamma ={\left\{\gamma_{t}\right\}}_{t=s+1}^{n};h={\left\{h_{t}\right\}}_{t=s+1}^{n}$). Then sampling is conducted for Φ and γ using the analog filter. The last step is the conduction of sampling for *h* by building a rational state space model; meantime, the sampling for *h*is completed using the moving method.

MCMC Algorithm

Given that $\Phi ={\left\{\Phi_{\text{t}}\right\}}_{\text{t }=\;1}^{\text{n}}$ and ω = (Σ_Φ_, Σ_γ_, Σ_*h*_) [the priori probability density is π(ω)], we can generate the samples from π(ϕ, γ, *h*|Φ|) based on the MCMC algorithm, with the value of Φ given. We use the MCMC algorithm as follows:

① Initialize Φ, γ, *h*, and ω;
② Take samples for Φ*|* γ, *h*, Σ_Φ_, and *y*;
③ Take samples for ∑ϕ|ϕ;
④ Take samples for γ*|* Φ, *h*, Σ_γ_, and *y*;
⑤ Take samples for ∑ɤ|γ;
⑥ Take samples for *h*|Φ, γ, Σ_*h*_, and y;
⑦ Take samples for ∑*h*|*h*;
⑧ Return to step 2.

Step 2 and step 4 require the filter to complete, while step 6 requires the stochastic volatility in mean (SVM) model to complete.

Based on the LT-TVP-VAR model, the paper estimates the time-varying correlation coefficients, deriving the potential time-varying characteristics. The coefficient estimates are summarized and analyzed. The time-varying characteristics of the stochastic volatility of the key variables are summarized and analyzed. An impulse response analysis is also conducted. Then the empirical analysis findings are derived.

## Data

The paper builds a LT-TVP-VAR model with four variables, i.e., monetary policy, EPU, output gap, and inflationary gap. Both quantitative monetary policy and price-based monetary policy are studied. The paper adopts the approach suggested by Baker et al. (19) by using big data technology to build EPU based on such mainstream media keywords as economy, policy, tax, and uncertainty. In order to improve data smoothness, the logarithm of EPU is worked out and seasonally adjusted using the X-12 method. The relevant data are obtained from the website of Economic Policy Uncertainty.

**Figure 1:**
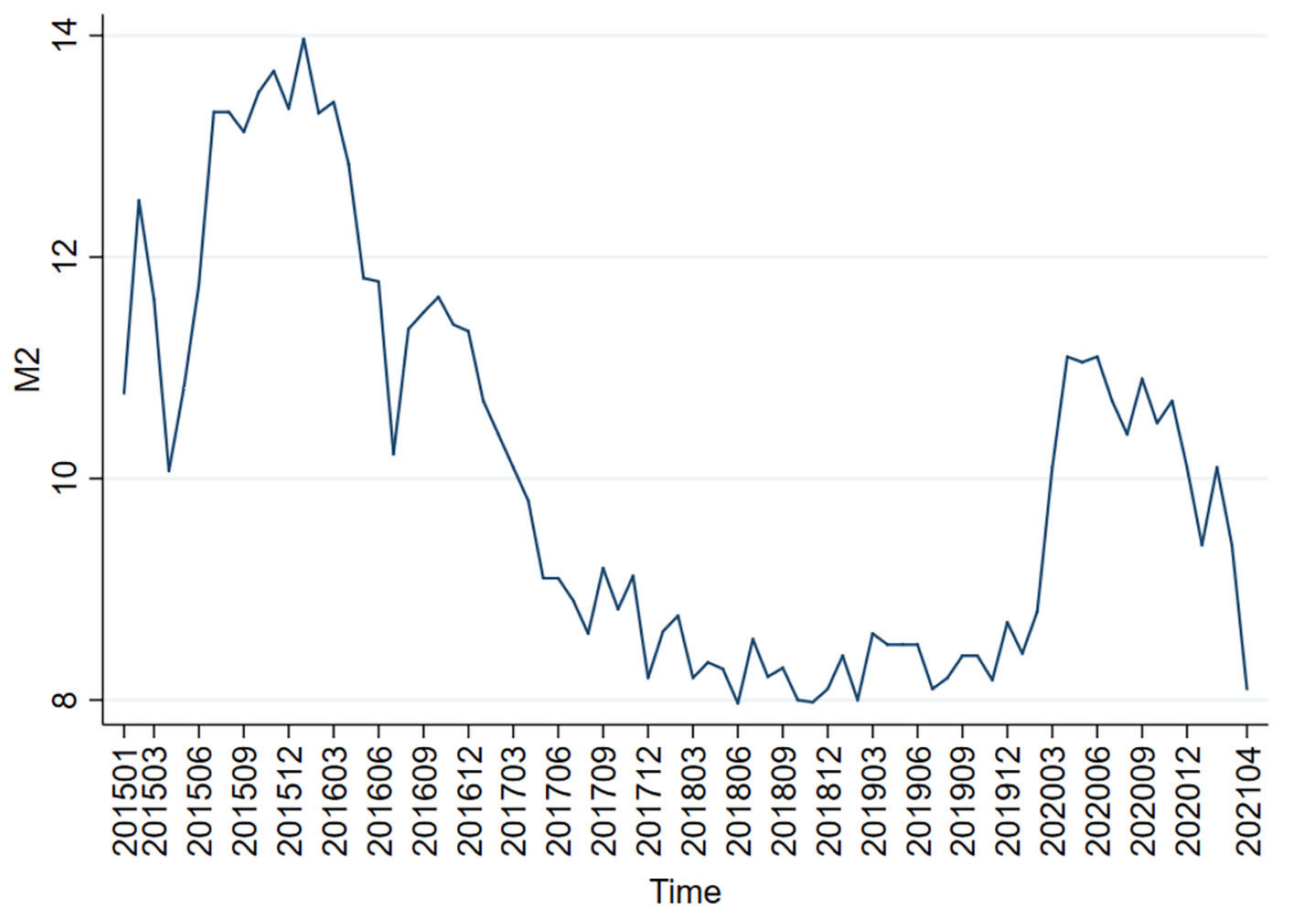
Trend chart of the QOQ-EOP M2 stock real growth rate.

**Figure 2:**
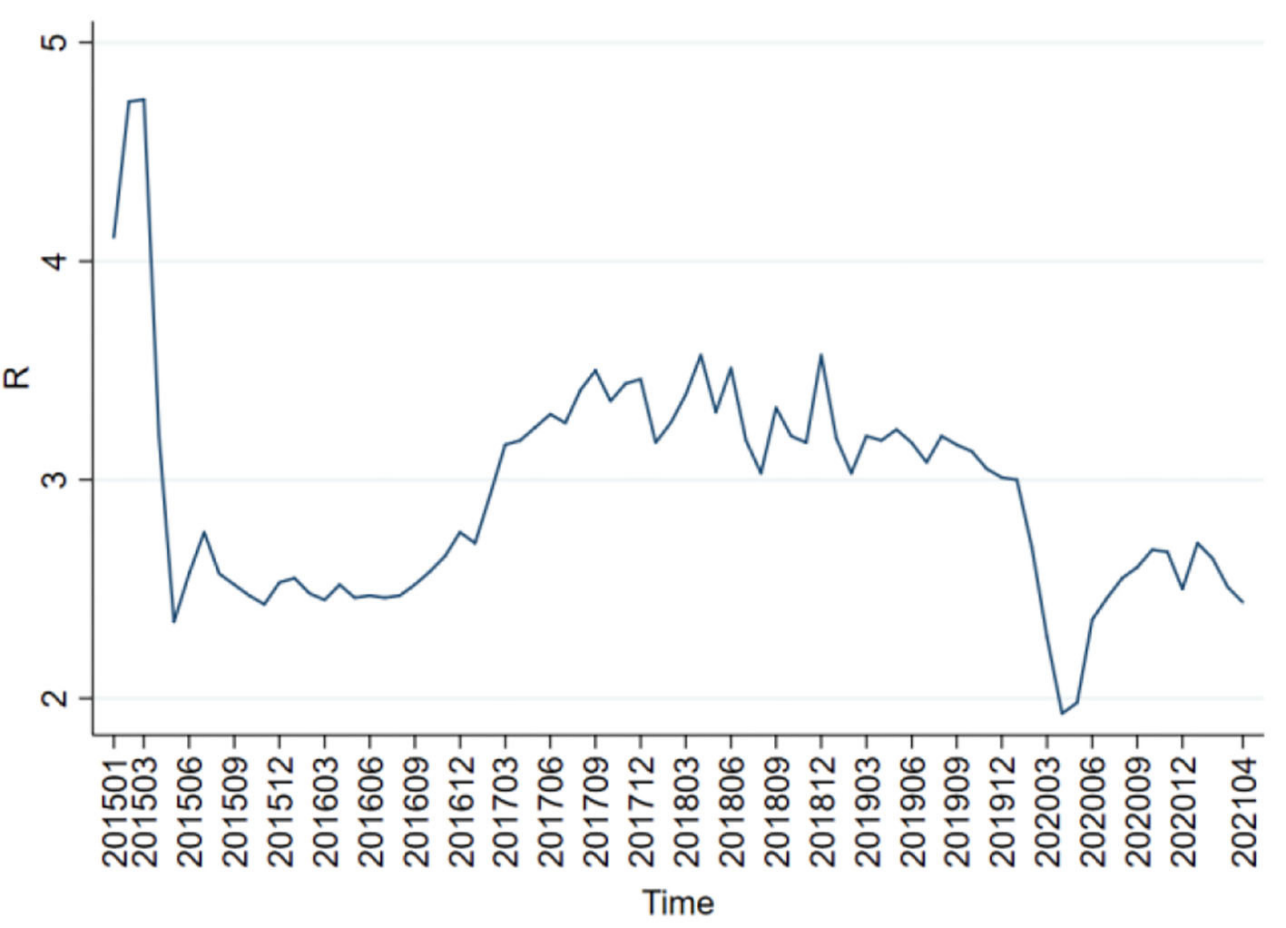
Trend chart of the weighted average of the 7-day interbank rate.

It is very important to compare the adjustment effects of quantitative monetary policy and price-based monetary policy because China is at the critical moment of switching from the former one to the latter one. Therefore, the month-over-month (MOM) end-of-period (EOP) M2 money stock real growth rate is selected as the proxy variable of quantitative monetary policy, and the weighted average of the 7-day interbank rate as the proxy variable of price-based monetary policy. Figures 1, 2 show the trends of the MOM-EOP M2 stock real growth rate and the weighted average of the 7-day interbank rate from January 2015 to April 2021, respectively. All the data are sourced from CEInet Statistics Database and government-issued figures. The sample data in the model are extracted from a monthly time series from January 2015 to April 2021.

**Figure 3:**
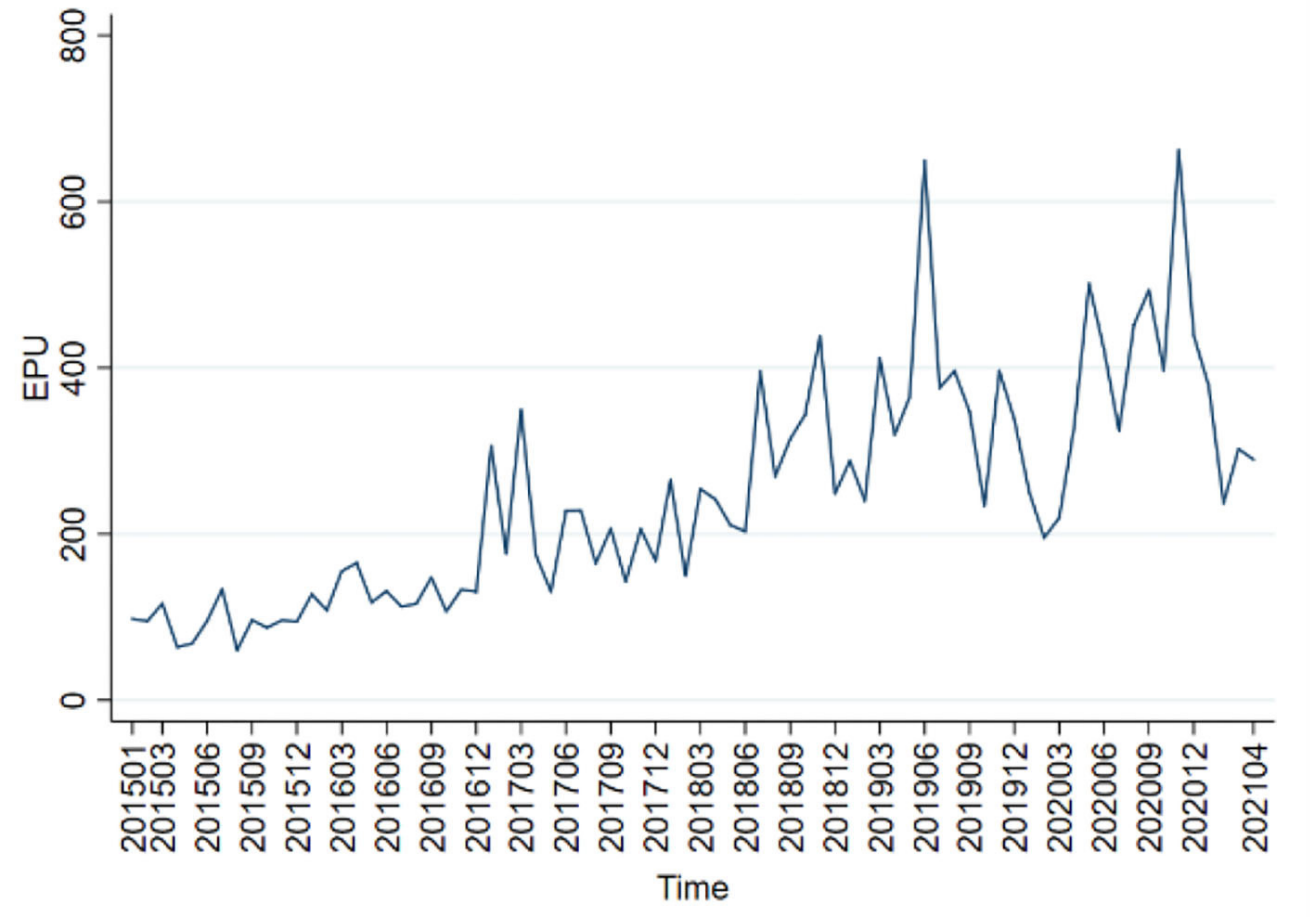
EPU trend chart.

By definition, EPU means the uncertainties of internal and external factors which an economy is subject to, e.g., macroeconomic environment and institution. The general trend and fluctuation of EPU are highly coupled with economic policy environment and external shock. The average level of EPU also differs at different stages. Figure 3 provides China's EPU trend from January 2015 to May 2021.

**Figure 4:**
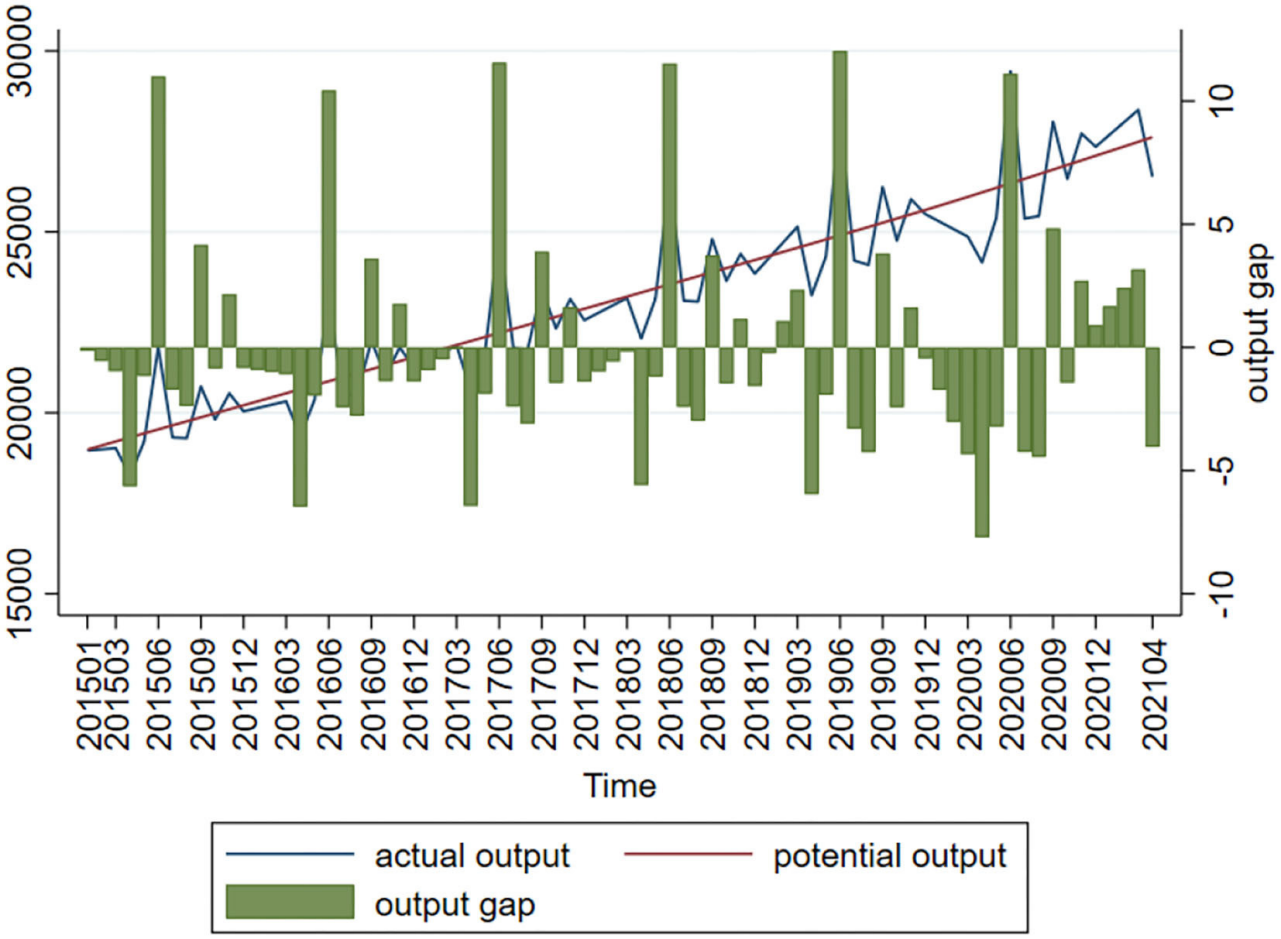
Output gap chart.

GDP is a very useful in measuring output. However, the Chinese government releases data only on a quarterly and annual basis, so these need be processed in order to obtain monthly data. Since converting quarterly or annual GDP into monthly GDP compromises the characteristics of raw data and therefore the scientificity of the findings, the method suggested by Chen and Sun (20) is used as a basis for calculating output gap. The industry value added (IVA) of businesses above designate size is used as the proxy variable of output. Based on the current MOM real growth rate, the real IVAs of the months of 2005 can be converted into the IVAs of the months and years, which represent the real output. The missing values are interpolated, and the H-P filter is used to extract the IVA trend components of the months and years as potential output (y^*^_t_). The last step is calculating output gap ($\bar{y}$) based on the formula y_t_ = 100 × ln(y_t_/y^*^_t_) (Figure 4 presents the results). In Figure 4, the column stands for output gap, the solid line stands for actual output and dotted line stands for potential output. Actual output and potential output are highly coupled in trend. Output gap always fluctuates around the zero line at different periods of time. As supply-side structural reform keeps going, the Chinese economy is transitioning from high-speed development to high-quality development. By and large, output gap has a small absolute value and assumes a stationary trend. Although China's economic sustainability has not turned generally negative in the long term, the downtrend risk is not to be neglected. In this context, the government must continue to step up efforts to control risk.

**Figure 5:**
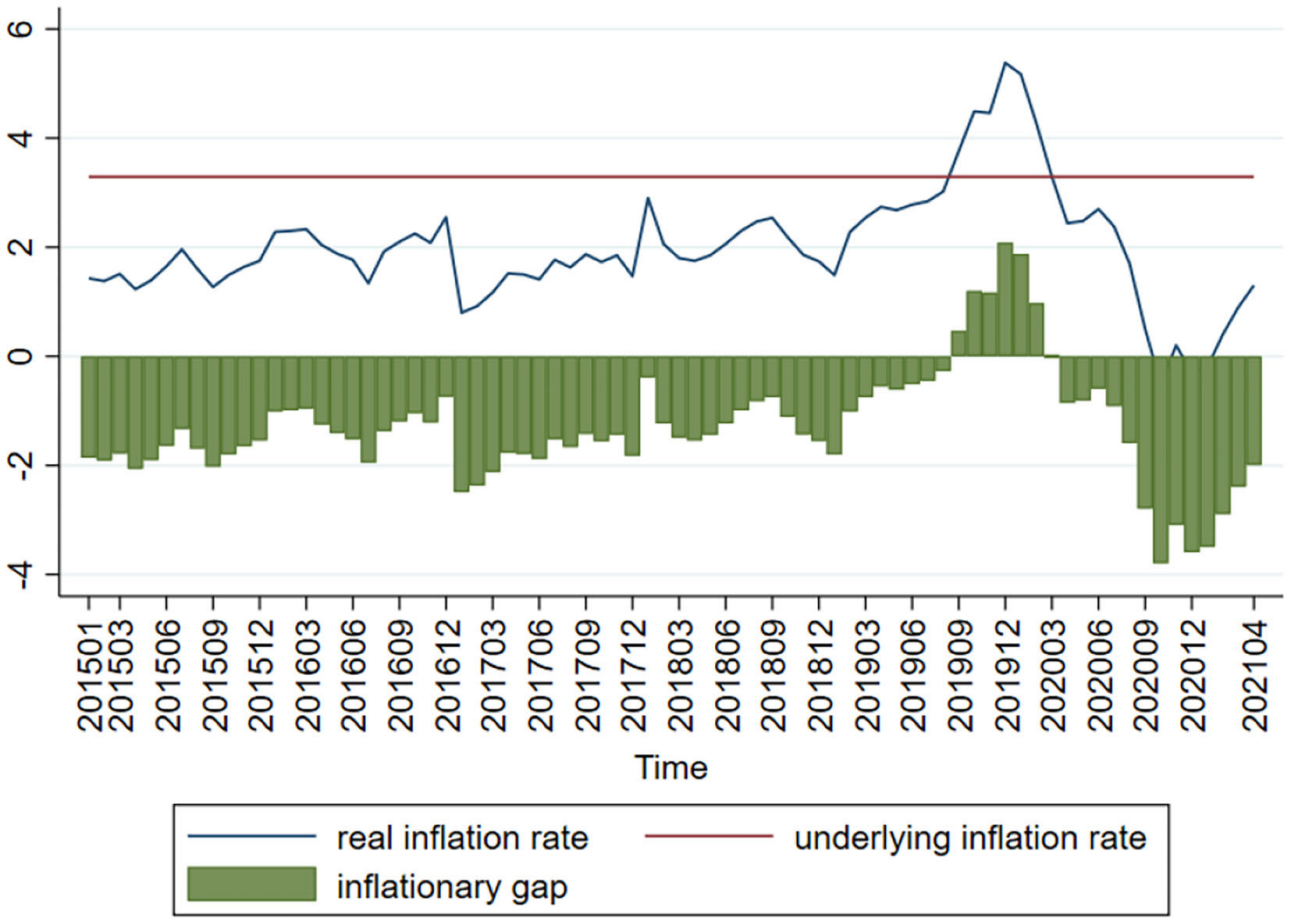
Inflationary gap chart.

Based on the approach of Liu and Xie (21), the current consumer product index (CPI) is used as the proxy variable of the real inflation rate. The targeted CPI increase control value is then taken out of the government-issued annual reports in order to calculate the targeted inflation rate. The average of the 2015–2021 targeted CPI increase control values is taken as the value of potential inflation rate in order to work out inflationary gap (or QCPI; see Figure 5). In Figure 5, the column stands for inflationary gap, the solid line stands for the actual inflation rate and the dotted line stands for the potential inflation rate. As with output gap, inflationary gap fluctuates around the zero line. The difference is that most of the time, inflationary gap in the sample interval deviates downwards from the zero line. It was suggested by Liu and Xie (21) that as China transitions from a fast-growing economy to a high-quality economy, inflationary gap will continue to deviate downwards from the zero line. Generally speaking, the Chinese government prefers to avoid inflation since very high inflation causes government credibility loss, asset price mismatch, welfare loss and a series of problems; meantime, very low inflation increases corporate running cost and profit shrinkage, leading to economic disorder.

The paper includes an augmented Dickey-Fuller (ADF) test as a unit root test of all sample data adopted prior to an empirical analysis. The unit root test results show that all variables other than output gap are non-stationary and pass the stationarity test after first-order differentiation (see Table 1). Therefore, the paper uses the price-based monetary policy instrument and quantitative monetary policy instrument as proxy variables, respectively, to build the time-varying parameter vector autoregression with stochastic volatility (TVP-VAR-SV) model. Model 1 adopts money supply, output gap, inflationary gap, and EPU as variables, while Model 2 adopts the weighted average of 7-day interbank rate, output gap, inflationary gap, and EPU as variables. Besides, the two data groups undergo the Johansen cointegration test whose results indicate that the two groups are co-integrated (see Table 1).

**Table 1:** ADF test results of the variables.

**Variable**	**Student's *t*-test**	**Critical value at 1% significance**	**Critical value at 5% significance**	**Critical value at 10% significance**	***P*-value**	**Conclusion**
EPU	−3.203	−3.552	−2.914	−2.592	0.0198	Non-stationary
D(EPU)	−13.789	−3.562	−2.920	−2.595	0.0000	Stationary
QCPI	−1.827	−3.553	−2.915	−2.592	0.3671	Non-stationary
D(QCPI)	−5.370	−3.563	−2.920	−2.595	0.0000	Stationary
Y	−9.927	−3.553	−2.915	−2.592	0.0000	Stationary
M2	−1.449	−3.552	−2.914	−2.592	0.5585	Non-stationary
D(M2)	−8.392	−3.562	−2.920	−2.595	0.0000	Stationary
R	−2.404	−3.552	−2.914	−2.592	0.1406	Non-stationary
D(R)	−6.202	−3.562	−2.920	−2.595	0.0000	Stationary

## Empirical Results

This paper uses the MCMC algorithm to estimate the correlated variables. The Akaike information criterion (AIC) minimum value is used and it shows that the model has a second-order lag. Tables 2, 3 show the parameter estimates of quantitative monetary policy and price-based monetary policy, respectively, based on 20,000 simulation trials.

**Table 2:** Parameter estimates of the quantitative model.

**Variable**	**Average value**	**Standard deviation**	**95% confidence interval**	**Geweke**	**Inef**
sb1	0.0230	0.0029	[0.0183, 0.0296]	0.128	3.33
sb2	0.0228	0.0026	[0.0183, 0.0285]	0.351	3.00
sa1	5.8412	82.5035	[0.0250, 32.4765]	0.056	11.50
sa2	0.0489	0.0655	[0.0291, 0.0926]	0.648	33.30
sh1	0.2618	0.0988	[0.1123, 0.4918]	0.249	32.53
sh2	0.7193	1.0014	[0.0949, 4.6281]	0.015	192.95

**Table 3:** Parameter estimates of the price-based monetary policy model.

**Variable**	**Average value**	**Standard deviation**	**95% confidence interval**	**Geweke**	**Inef**
sb1	0.0228	0.0027	[0.0183, 0.0288]	0.991	13.20
sb2	0.0228	0.0026	[0.0183, 0.0287]	0.709	4.00
sa1	1.4381	11.0802	[0.0238, 14.3306]	0.191	58.91
sa2	0.0455	0.0391	[0.0291, 0.0814]	0.036	41.05
sh1	0.2698	0.1033	[0.1201, 0.5216]	0.648	44.67
sh2	0.4698	0.3597	[0.0758, 1.4044]	0.785	254.27

It can be seen from the Geweke values in Tables 2, 3 that the MCMC algorithm has a good simulation effect. All Geweke values are smaller than the critical value (1.96) at a 5% significance level, which means that the 20,000 simulation trials are successful in generating an adequate effect sample size. Besides, the maximum value of the ineffective shock factor is 192.95 in the quantitative model, which means that at most 104 (2,00,00/192.95) uncorrelated samples are generated. The maximum value of the price-based model is 254.27, which means that at most 79 (2,00,00/254.27) uncorrelated samples are generated.

Table 4 shows the potential threshold values of the quantitative model. The acceptability is >40% for all parameters, indicating a threshold relationship between the models' correlated variables. The acceptability, respectively, are 85.6 and 41.3% for parameters (d_α_)_1_ and (d_α_)_2_ in the quantitative model, while the acceptability, respectively, are 81.9 and 46.2% for parameters (d_α_)_1_ and (d_α_)_2_ in the price-based model. It demonstrates that the highly-fluctuating parameters are smoothed for both models during the simulation. Also, there is a significant threshold relationship between the variables of the models.

**Table 4:** Potential parameter threshold value acceptability (%) of the quantitative model and the price-based model.

	**Quantitative model**		**Price-based model**	
Parameter	(d_α_)_1_	(d_α_)_2_	(d_α_)_1_	(d_α_)_2_
Acceptable rate	85.6	41.3	81.9	46.2

**Figure 6:**
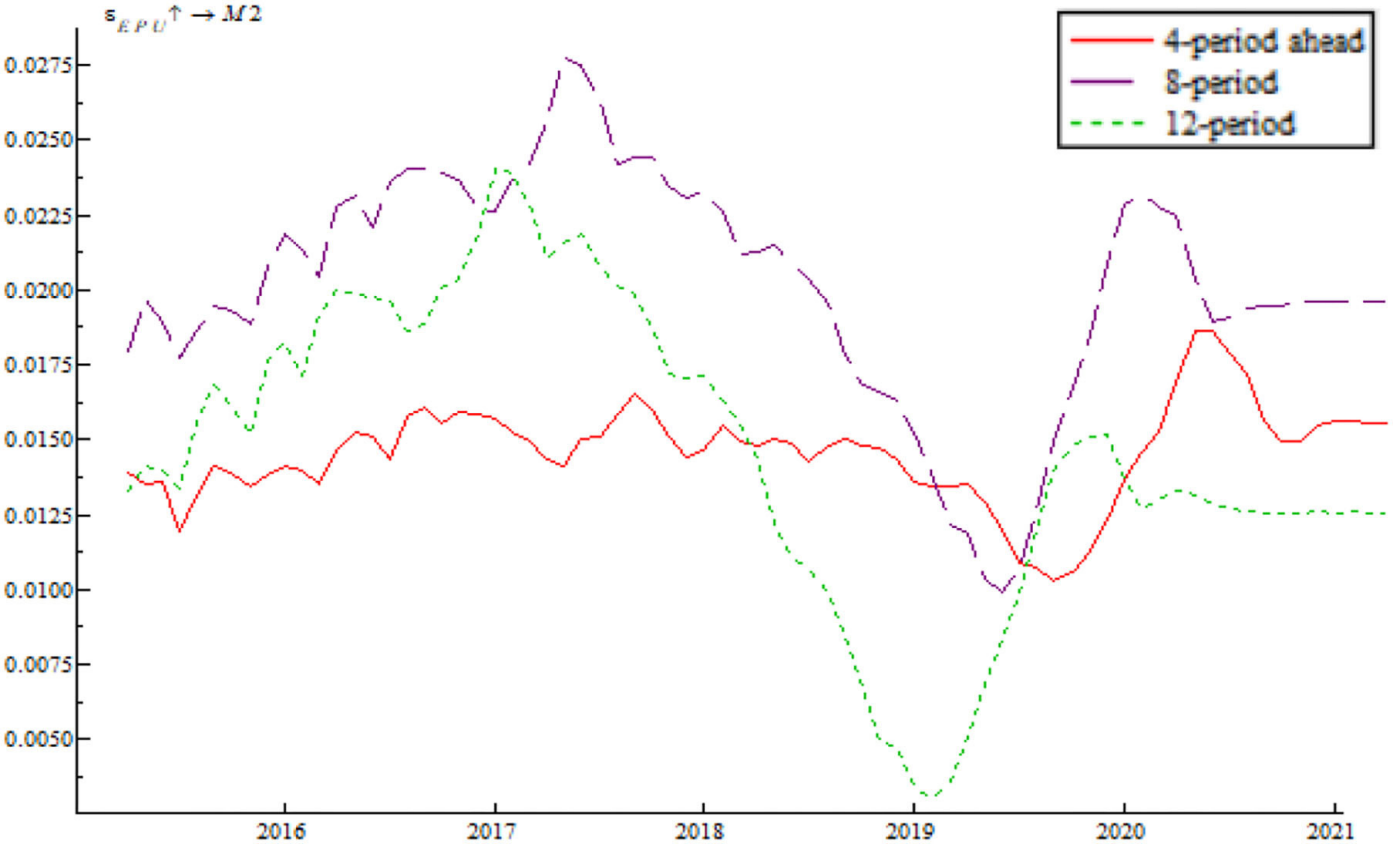
EPU's shock on quantitative monetary policy.

**Figure 7:**
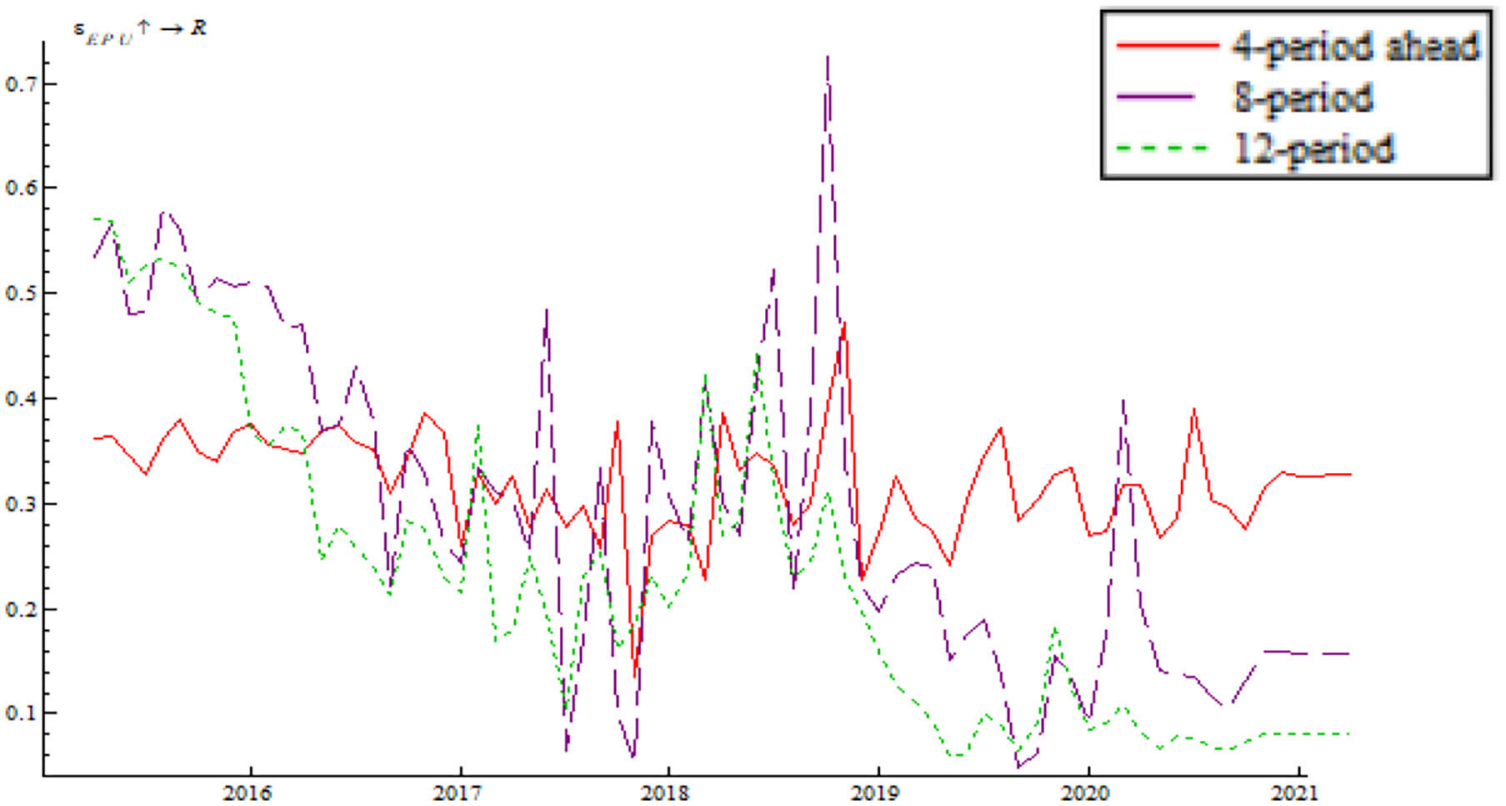
EPU's shock on price-based monetary policy.

Based on the time-varying impulse response function (tvIRF) of the LT-TVP-VAR model, we can show the relationship between EPU's shock and the monetary policy effectiveness. The paper considers short-term effect, mid-term effect and long-term effect for the dynamic shock of EPU on monetary policy dynamic as 4-, 8-, and 12-month shocks, respectively, all in terms of cumulative impulse response. According to Figures 6, 7, the short-term, mid-term, and long-term curves assume a generally consistent trend on the whole, demonstrating that the calculations of the LT-TVP-VAR model are robust to a certain degree. EPU is basically positively correlated with the short-term, mid-term, and long-term effects of the quantitative monetary policy and price-based monetary policy. It means that EPU only influences the magnitude of monetary policy effectiveness. The coefficient of EPU's shock on monetary policy effectiveness is basically between 0 and 1. Besides, when EPU is higher, the coefficient is nearer to 0, indicating that the EPU's shock will drive down monetary policy effectiveness. In addition, the mid-term lag function (stage 8) and long-term lag function (stage 12) are basically above the short-term lag function (stage 4); that is to say, EPU has a stronger short-term shock on quantitative monetary policy and price-based monetary policy.

As shown in Figures 6, 7 we find that the impulse function of EPU's shock on quantitative monetary policy assumes an upward trend and that the impulse function of EPU's shock on price-based monetary policy assumes a W-like trend, indicating that the central bank used multiple price-based monetary policy instruments to achieve goals as expected. In spite of such serious risks associated with deleveraging and asset bubble, China persisted in robust monetary policy by reducing RMB base rate. In addition, the central bank achieved its anticipated goals through open market regulation, mid-term lending facilities, reserve loan facilities, and other financial tools. From 2018 onwards, the impulse function of EPU's shock on quantitative monetary policy and price-based monetary policy dropped to around the zero sharply, bottomed out and rose again near the zero line, which is due to the 2018 China-U.S. trade dispute. The subsequent rebound is attributable to the Chinese central bank's efforts to mitigate the trade war shock by reduction in required reserve, and open market transaction. From 2020 onwards, the impulse function of EPU's shock on quantitative monetary policy and price-based monetary policy fell again due to the COVID-19 pandemic. However, the second fall is smaller than during the 2018 trade war, indicating the central bank's improvement in responding to uncertainty.

**Figure 8:**
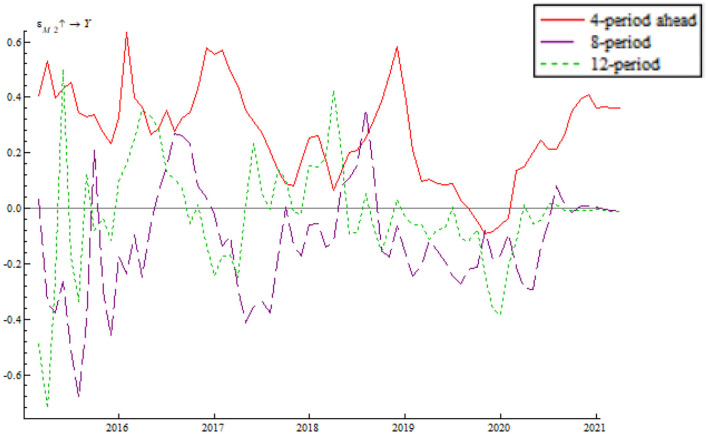
Shock of the quantitative monetary policy instrument on output gap.

As shown in Figure 8, the impulse function of the shock of quantitative monetary policy takes a W-shaped trend, fluctuating around the zero line. Meanwhile, the fluctuation range decreases with time, indicating that the Chinese central bank manages to use multiple quantitative monetary policy instruments to keep output gap within a reasonable range. In spite of the 2018 China-U.S. trade war and other uncertainty factors, the central government began tightening monetary policy at the end of 2018 while putting more importance on other counter-cyclical measures. Examples include a series of required reserve reduction efforts, flexible open market regulation, mid-term lending facilities, which combine to hedge against the shock of uncertainties and maintain economic resilience. Immediately after the outbreak of the COVID-19 pandemic, the Chinese central bank issued and implemented quantitative easing (QE) policy as well as four direct policy instruments (special refinancing bond, rediscounting, moratorium on debt repayment, and credit support plan). These measures prevented a sharp rise in output gap.

**Figure 9:**
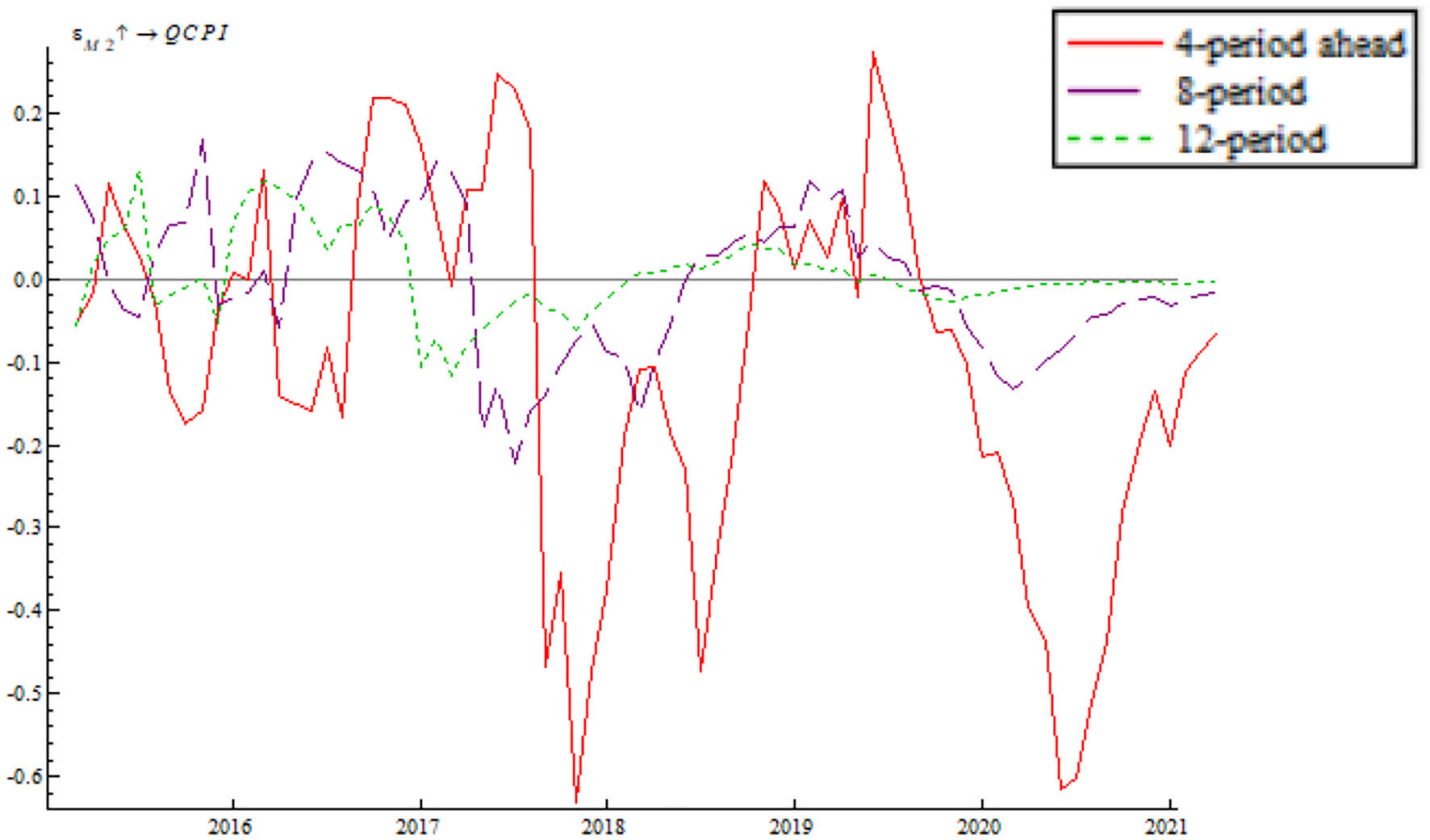
Shock of the quantitative monetary policy instrument on inflationary gap.

As shown in Figure 9, the impulse function of the shock of quantitative monetary policy on inflationary gap assumes a W-shaped trend. Before 2018 it fluctuated around zero line and from 2018 onwards, there was a primarily negative effect coupled with increased fluctuation. This is due primarily to the 2018 China-U.S. trade dispute and the 2020 pandemic aftershock. At the same time, it shows that the Chinese central bank needs to improve the capability of using the quantitative monetary policy instrument to maintain effective demand and keep price level stable. In both 2018 and 2020 there was a trough and a negative inflationary gap, indicating that the positive shock of the quantitative monetary policy instrument relieved the deflation to some degree. The 2018 trade war as well as the 2020 pandemic aftershock increased supply pressure. In response to the deflation, the central bank adopted prudent monetary policy to adjust monetary aggregates counter-cyclically. The 2019 crest indicates that the positive shock of the quantitative monetary policy instrument further intensify inflation. This is because increasing agricultural product price level due to the China-U.S. trade war as well as international hot money inflow.

**Figure 10:**
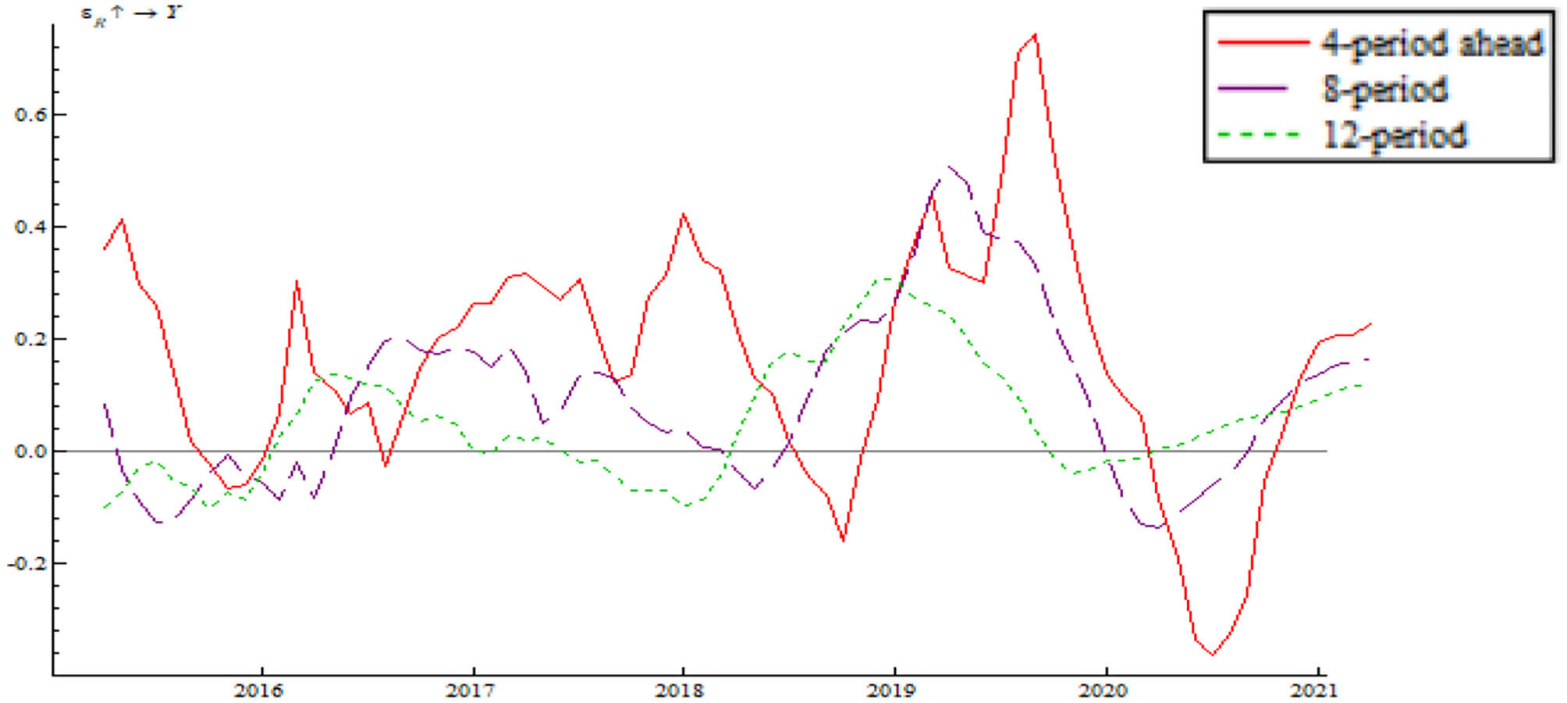
Shock of price-based monetary policy on output gap.

As shown Figure 10, the shock of price-based monetary policy on output gap assumes a W-shape trend which fluctuates around the zero line, which indicates that the central bank can make counter-cyclical adjustment using the price-based monetary policy instrument. In response to the negative output gap resulting from the China-U.S. trade war in 2018, the central bank implemented multiple rounds of reduction in the interest rate. The measure brought about a positive output gap as well as a crest in 2019. This is attributable to the economic downturn pressure resulting from the China-U.S. trade friction. The central bank improved the loan prime rate (LPR) mechanism in order to break through the lower bound of interest rate and introduce the mid-term lending facility to make the determination of interest rate more market-based. The central bank also took advantage of macro-prudential counter-cyclical adjustment to lead the market, stabilize market confidence, and increase liquidity. In 2020, the impulse function of the shock of price-based monetary policy on output gap rebounded quickly after bottoming out. This is because the central bank introduced loose monetary policy immediately after the outbreak of the pandemic. Price-based monetary policy was adopted for several times, successfully increasing money velocity quickly, driving market demand upsurges, and causing a rebound in the wake of the trough.

**Figure 11:**
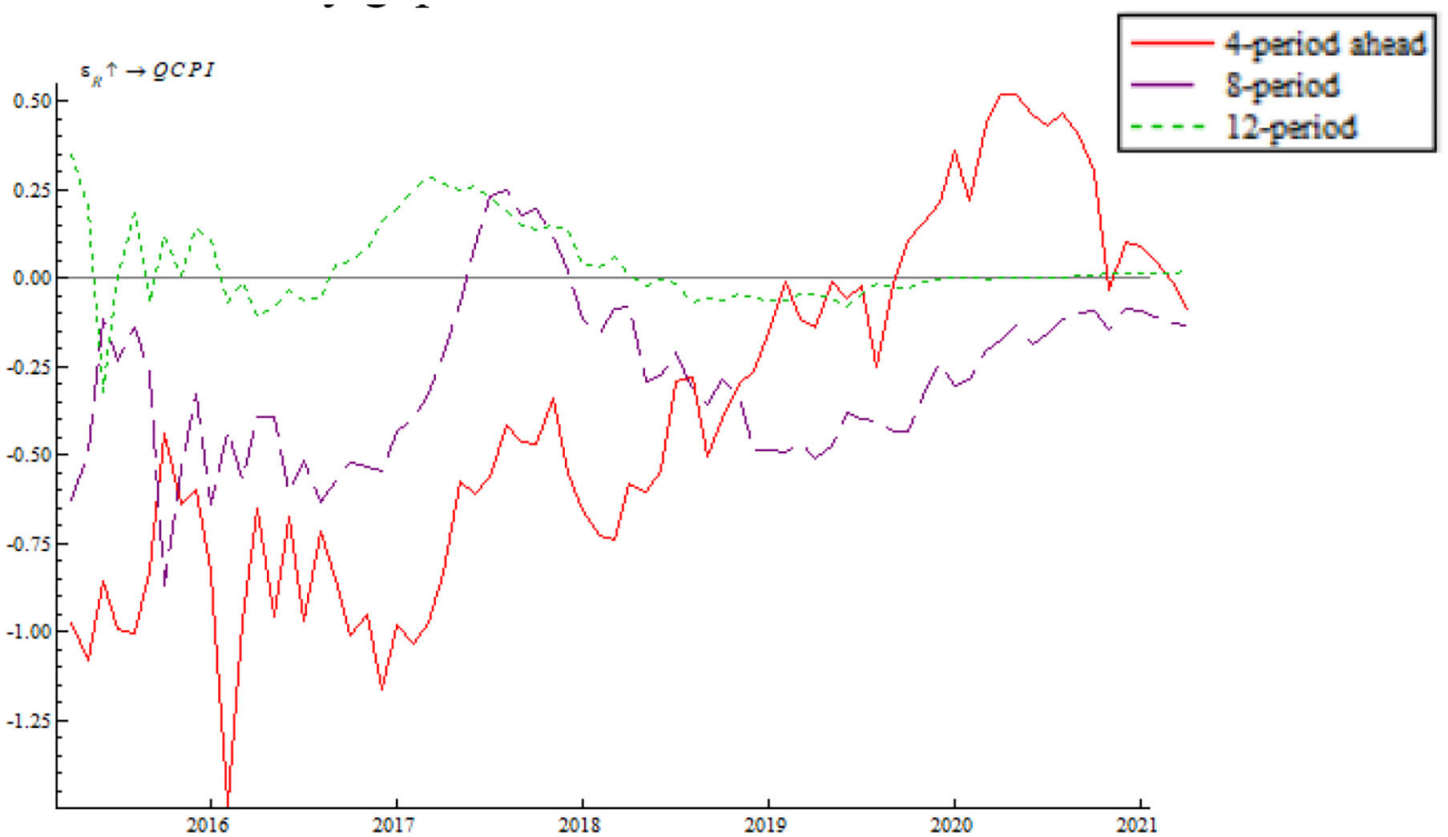
Shock of the price-based monetary policy instrument on inflationary gap.

As shown in Figure 11, the shock of price-based monetary policy on inflationary gap makes largely a negative effect, indicating that the positive shock of the interest rate per unit of standard deviation can narrow inflationary gap; besides, it means that the interest rate has a good counter-cyclical adjustment effect on the price level and that the central bank will avoid using overly tight monetary policy in price adjustment. Under the influence of the slow and unstable recovery of the global economy, inflationary gap dipped to trough for the sampling period. Hence, the central bank eased monetary policy by lowering the base rate and introducing expansionary fiscal policy. These measures led to a quick rebound after inflationary gap bottomed out. The shock of 2017–2018 price-based monetary policy on inflationary gap assumed an uptrend; at the same time, the impulse function decreased in 2018. This is because the China-U.S. trade war exerted negative impact on China's foreign trade and food price surged. The central bank implemented a series of flexible counter-cyclical adjustments. As a result, inflation rate was well-controlled and the impulse function changed once again from a decrease to an increase. In 2020, the impulse function again decreased; as a response the central bank adopted adequate preventive monetary policy by reducing the nominal rate and leveraging the interest rate to increase money velocity and narrow inflationary gap.

**Figure 12:**
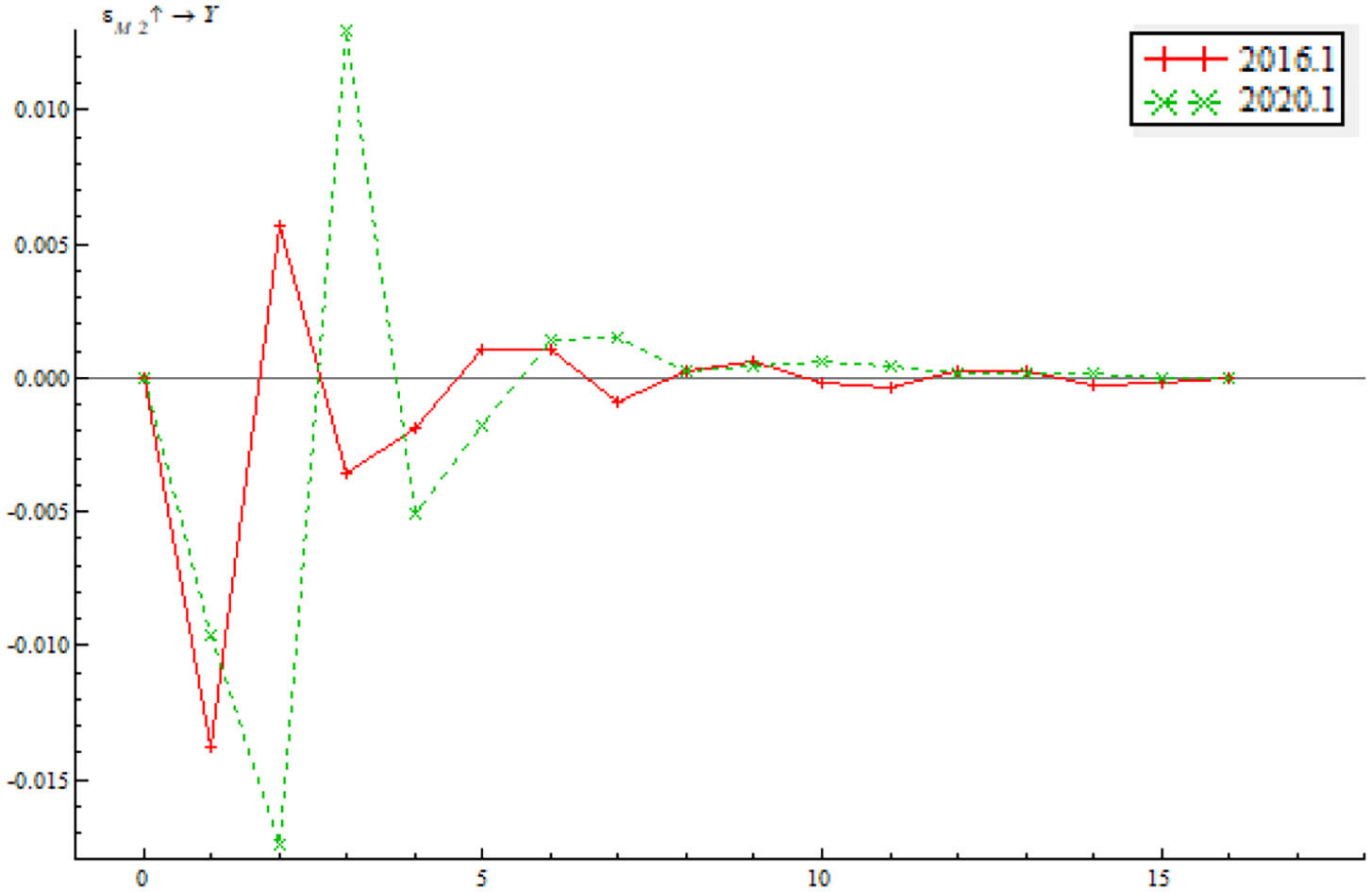
Time point-based shock of quantitative monetary policy on output gap.

The paper selects a sample period of January 2016 through January 2020 for an investigation on what role that quantitative monetary policy and price-based monetary policy played in a dynamic adjustment to output gap and inflationary gap before and after the pandemic. The sample period contains both low EPU and high EPU. Through a comparative analysis of the impulse response function between pre-pandemic and post-pandemic, we can find the path by which monetary policy adjusts to output gap and inflationary gap as well as the time-varying characteristics in the context of EPU. As shown in Figure 12, the time point impulse function of the shock of quantitative monetary policy on output gap proves negative in the short term. In the wake of a change in the positive shock between stage 2 and stage 4, there was a short but minor negative shock. From stage 8 onwards, the impulse function gradually inclined to zero, indicating that the shock of quantitative monetary policy on output gap last 2–4 stages. While the trends of the two impulse functions are largely consistent, the shock of quantitative monetary policy on output gap is greater in January 2020 than January 2016. This indicates that the shock of the quantitative monetary policy instrument on output tends to last longer as EPU increases. Besides, the shock of the quantitative monetary policy instrument on output gap increases with a growth in EPU.

**Figure 13:**
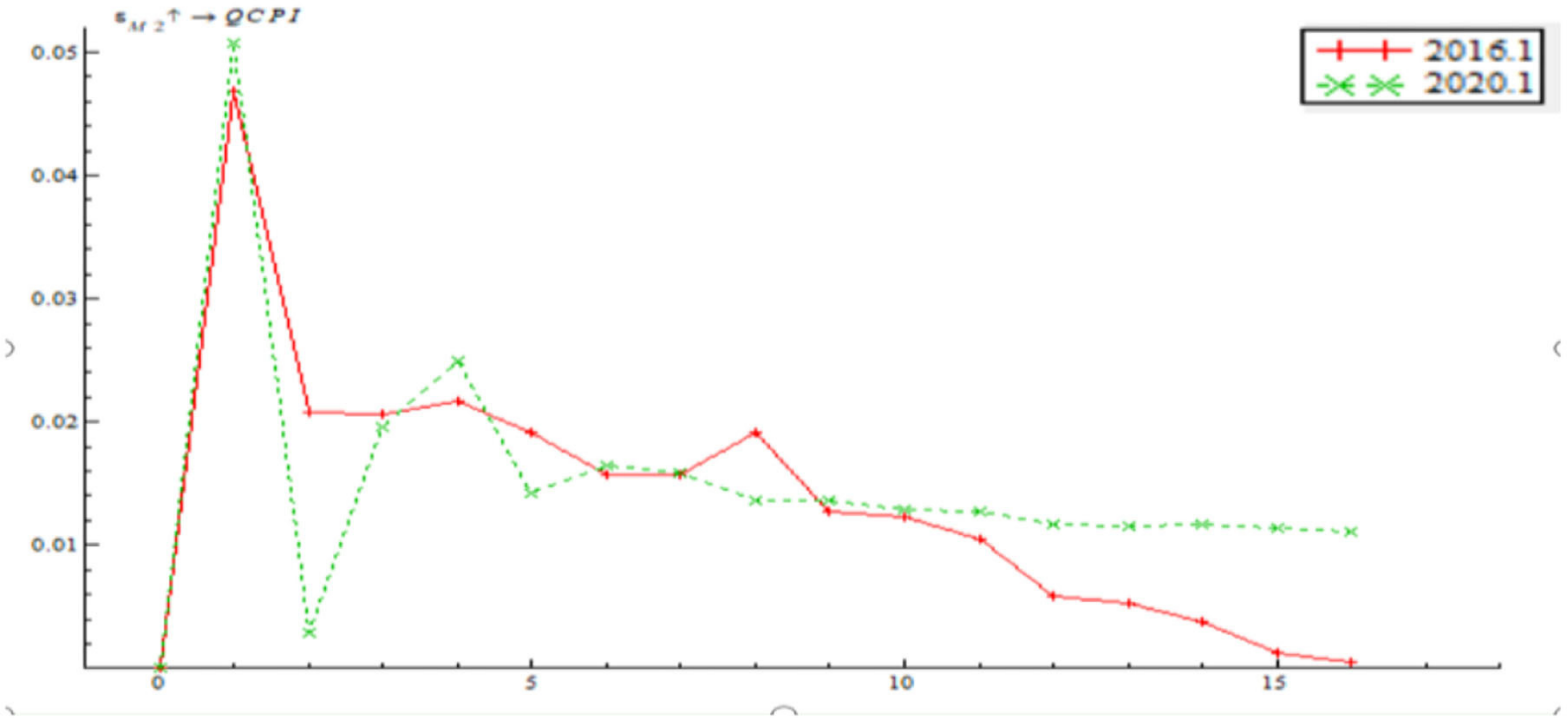
Time point-based shock of quantitative monetary policy on inflationary gap.

As shown in Figure 13, quantitative monetary policy has a positive shock on inflationary gap on the whole, indicating that if money supply increases at the time, the price level will climb. The shock in January 2020 is basically consistent with that in 2016, indicating that the central bank improves the ability to use the quantitative monetary policy instrument for inflation adjustment. The difference is that the duration of the shock in January 2020 is obviously longer than in January 2016, indicating that the shock will increase as EPU climbs. This is partly due to the formulation and implement of the central bank's monetary policy in response to the pandemic.

**Figure 14:**
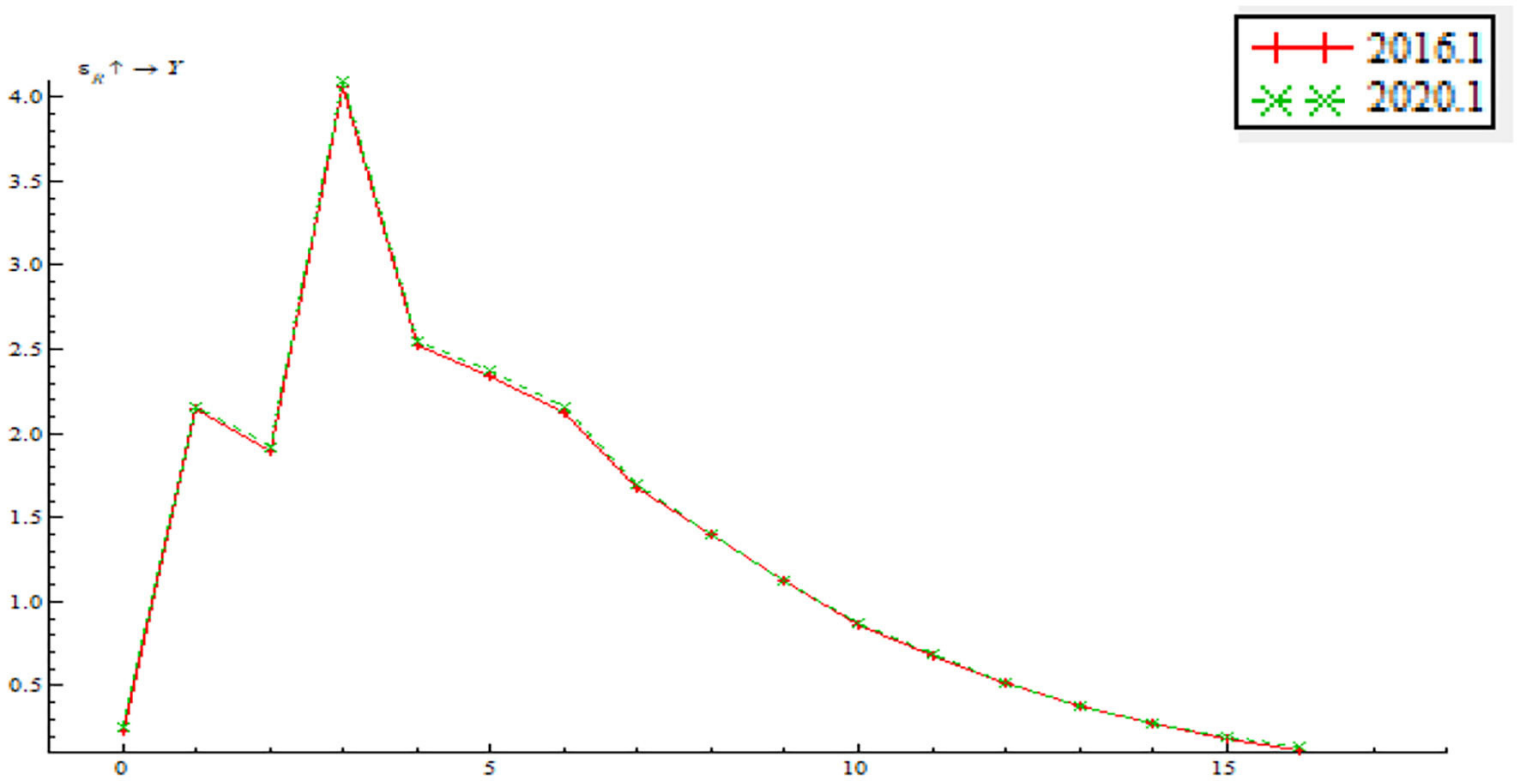
Time point-based shock of price-based monetary policy on output gap.

As shown in Figure 14, price-based monetary policy has a positive shock on output gap; that is to say, interest rate rise tends to make output gap bigger. The price-based monetary policy instrument mainly involves nominal rate adjustment. Judging from model specification, this means that higher EPU results in a greater shock of the price-based monetary policy on output gap. In other words, when EPU increases, price-based monetary policy instruments, such as interest rate reduction, can narrow output gap and realize stable economic growth.

**Figure 15:**
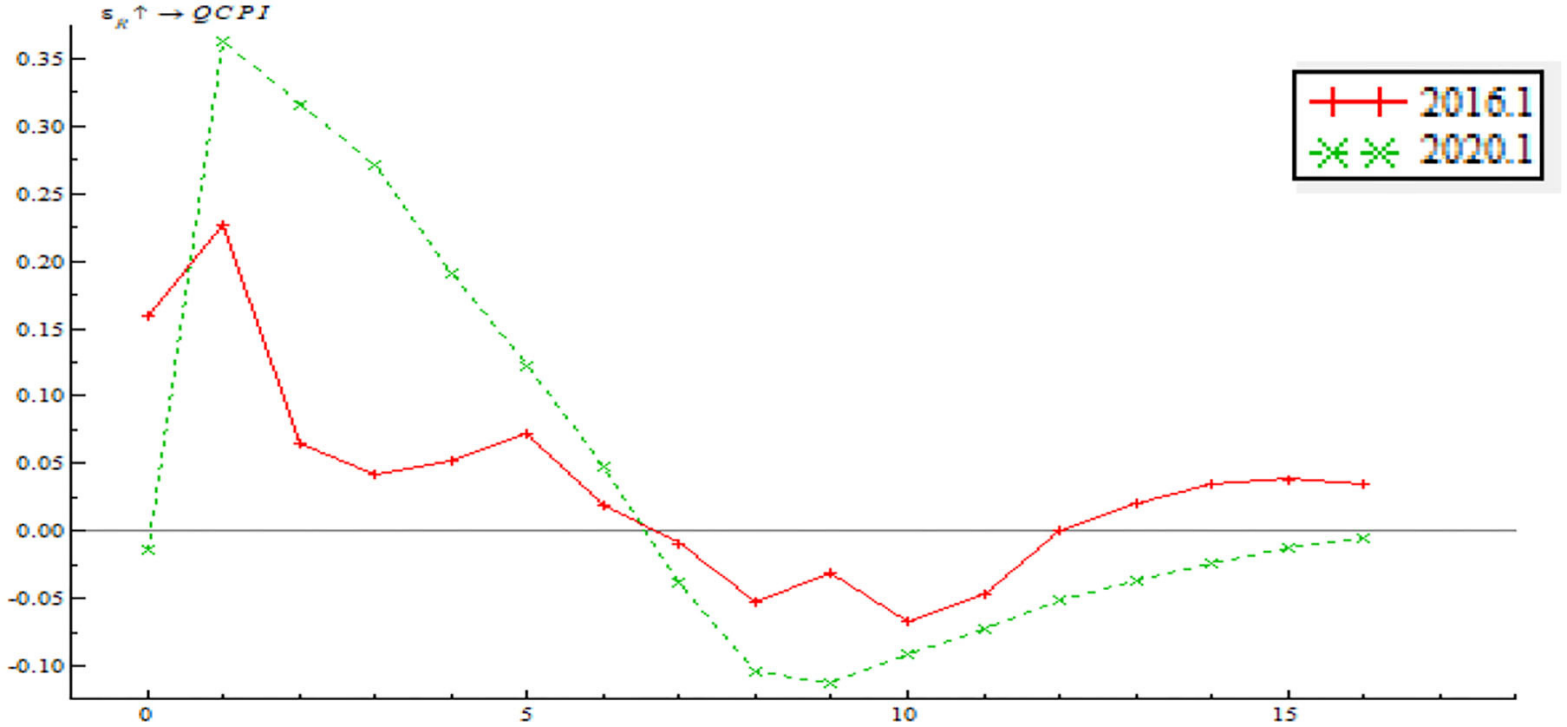
Time point-based shock of price-based monetary policy on inflationary gap.

As shown in Figure 15, the time point impulse functions of price-based monetary policy on output gap are largely consistent and the impulse response function mostly has a positive effect. After stage 7, the impulse function has a negative effect, indicating that the price-based monetary policy instrument has yet to be improved significantly and that the central bank should put importance on the long-term effect of regulatory policy as well. On the whole, as EPU increases, the price-based monetary policy has an increasingly significant shock on inflationary gap; that is to say, the interest rate policy has an increasingly significant shock on inflationary gap. Besides, the impulse function in June 2020 staggered before a gentle drop, which is attributable to multiple anticipatory steps taken by the central bank in response to the shocks of the pandemic, such as lending rate reduction, partial required reserve reduction, MLF, and LPR mechanism improvement. These steps serve to stimulate consumption, contribute to steady economic growth and limit price fluctuation within a reasonable range. This also signifies a significant improvement in the central bank's ability to address inflation using price-based monetary policy.

## Conclusion

The paper begins by building the LT-TVP-VAR model to investigate the dynamic mechanism that links EPU to quantitative monetary policy and price-based monetary policy. Then it investigated how the quantitative monetary policy instrument and the price-based monetary policy instrument affect output gap and inflationary gap. Finally, a comparative analysis, based on the time point-based impulse response function, is conducted to analyze the time-varying effects of the different policy instruments on output gap and inflationary gap between pre-pandemic and post-pandemic periods which contain different levels of EPU.

The following interpretations and conclusions are made from the above empirical results. First, the impact of EPU on the quantitative monetary policy shock and the price-based monetary policy shock has a significant threshold effect. In addition, there exists a positive correlation between EPU and the two monetary policy instruments regarding the short-term, mid-term, and long-term effects, respectively. Second, the quantitative monetary policy instrument is more powerful in dealing with output than inflation. The implementation of quantitative monetary policy instrument by the Chinese central bank turns out to be counter-cyclical. However, the time varying effect of the quantitative monetary policy instrument is primarily negative, which indicates that the central bank has yet to improve its ability to use the monetary policy instruments fighting against output gap and growth in price level. Third, the effect of the price-based monetary policy instrument used by the Chinese central bank to stabilize output and price level turns out to be significant. Fourth, both the effect of quantitative monetary policy and that of price-based monetary policy are shown to be increasingly powerful on output gap as EPU grows. In a comparative analysis, the effect of quantitative monetary policy instrument on output gap is shown to be more significant in the mid-term and long-term than the short-term, which exhibits a lag effect; and the lag time gets longer as EPU grows. At different levels of EPU the effects of price-based monetary policy on output gap are all remarkable.

We further make the following recommendations. First, the EPU index that is based only on *South China Morning Post* needs to be improved for more accuracy. Researchers can collect information from additional sources and channels. Second, the government should promote effective disclosing of information to the public to lower the level of EPU. Third, a combination of various monetary policy instruments can improve monetary policy's counter-cyclical effect. Particularly, price-based monetary policy instrument turns out to be more powerful in both output and price level. Therefore, the central bank should continue to improve the interest rate transmission mechanism and put more weight on price-based monetary policy than on quantitative monetary policy. In addition, when EPU is high, we need to combine quantitative monetary policy with price-based monetary policy in order to fight against output gap and inflationary gap pressure.

## Data Availability Statement

The original contributions presented in the study are included in the article/supplementary material, further inquiries can be directed to the corresponding author/s.

## Author Contributions

YS: conceptualization, methodology, formal analysis, writing—original draft preparation, and funding acquisition. YY: data processing, formal analysis, and writing—original draft preparation. JY: conceptualization, project management, writing—review and editing, and funding acquisition. ZZ: conceptualization and project management. All authors contributed to the article and approved the submitted version.

## Funding

The authors acknowledge the financial support from the National Social Science Foundation (the impact of heterogeneity of regional trade in services agreements on the reconstruction of global value chain of China's manufacturing industry; Project No: 20BJY091) and the innovation team project of Philosophy and Social Sciences in Colleges and Universities of Henan Province (coordinated development of urban and rural areas and Rural Revitalization; Project No: 2021-CXTD-04).

## Conflict of Interest

The authors declare that the research was conducted in the absence of any commercial or financial relationships that could be construed as a potential conflict of interest.

## Publisher's Note

All claims expressed in this article are solely those of the authors and do not necessarily represent those of their affiliated organizations, or those of the publisher, the editors and the reviewers. Any product that may be evaluated in this article, or claim that may be made by its manufacturer, is not guaranteed or endorsed by the publisher.

## References

[1] Bloom N Stephen B John V. Uncertainty and investment dynamics. Rev Econ Stud. (2007) 2:391–415. 10.1111/j.1467-937x.2007.00426.x

[2] Talavera O Tsapin A Zholud O. Macroeconomic uncertainty and bank lending: the case of Ukraine. Econ Syst. (2012) 36:279–93. 10.1016/j.ecosys.2011.06.005

[3] Li FY Yang MZ. Can economic policy uncertainty influence corporate investment? The empirical research by using China economic policy uncertainty index. J Finan Res. (2015) 4:115–29.

[4] Wu W Tiwari AK Gozgor G Leping H. Does economic policy uncertainty affect cryptocurrency markets? Evidence from Twitter-based uncertainty measures. Res Int Bus Finan. (2021) 58:101478. 10.1016/j.ribaf.2021.101478

[5] Song YG Hao F Hao XZ Gozgor G. Economic policy uncertainty, outward foreign direct investments, and green total factor productivity: evidence from firm-level data in China. Sustainability. (2021) 4:2339. 10.3390/su13042339

[6] Mccallum BT. Robustness properties of a rule for monetary policy. Carnegie Rochest Conf Ser Public Policy. (1988) 29:173–203. 10.1016/0167-2231(88)90011-5

[7] Taylor JB. Discretion versus policy rules in practice. Carnegie Rochest Conf Ser Public Policy. (1993) 195–214. 10.1016/0167-2231(93)90009-L

[8] Xu Z. Transformation of monetary policy in the high-quality development stage. J Finan Res. (2018) 4:1–19.

[9] Ma L Fan W. Research on the monetary policy regulation mechanism responding to the epidemic shock. Econ Sci. (2021) 2:19–32. 10.12088/PKU.jjkx.2021.02.02

[10] Bernanke BS Boivin J Eliasz P. Measuring the effects of monetary policy: a Factor-Augmented Vector Autoregressive (FAVAR) approach. Q J Econ. (2005) 1:387–422. 10.1162/0033553053327452

[11] Silvia M Giovanni R. The Transmission of Monetary Policy Shocks. The Warwick Economics Research Paper Series (TWERPS) (2017) p. 1136. 10.2139/ssrn.2957644

[12] Zhang L Jiang L. Orientation evolution and quantity-price transition of China's monetary policy regulation: also on the imitative effect of quantitative measurement of regulation orientation. Contemp Finan Econ. (2020) 9:52–65. 10.13676/j.cnki.cn36-1030/f.2020.09.006

[13] Zhuang ZG Cui XY Zhao XJ. Uncertainty, macroeconomic fluctuation and the choice of monetary policy rules in China: the quantitative analysis based on Bayesian DSGE model. Manag World. (2016) 11:20–31+187. 10.19744/j.cnki.11-1235/f.2016.11.003

[14] Pellegrino G. Uncertainty and the real effects of monetary policy shocks in the Euro area. Econ Lett. (2018) 162:177–81. 10.1016/j.econlet.2017.10.006

[15] Jin CY Zhang DY. The macro-adjustment and control effect of monetary policy under the condition of economic uncertainty in China. J Xi'an Jiaotong Univers. (2019) 2:1–11. 10.15896/j.xjtuskxb.201902001

[16] Talavera O Tsapin A Zholud O. Macroeconomic uncertainty and bank lending: the case of Ukraine, DIW Discussion Papers, No. 637, Berlin: Deutsches Institut für Wirtschaftsforschung (DIW), (2006). Available online at: http://hdl.handle.net/10419/18530

[17] Georgiadis G Mehl A. Financial globalization and monetary policy effectiveness. J Int Econ. (2016) 103:200–12. 10.1016/j.jinteco.2016.10.002

[18] Xu ZW Wang WF. Does policy uncertainty drive Chinese aggregate fluctuations? —Evidence and dynamic analysis. China Econ Q. (2019) 1:23–50. 10.13821/j.cnki.ceq.2018.02.02

[19] Baker SR Bloom N Davis SJ. Measuring economic policy uncertainty. Q J Econ. (2016) 4:1593–636. 10.2139/ssrn.2198490

[20] Chen SD Sun YL. The analysis of the output gap effect and risks of fluctuation in China's financial cycle. Finan Forum. (2018) 7:25–34. 10.16529/j.cnki.11-4613/f.2018.07.004

[21] Liu JQ Xie YZ. The characteristics of monetary policy changes and the choice of control modes in the period of “new normal”. J Finan Res. (2016) 9:1–17.

